# Improvement of near-infrared (NIR) reflectivity and black color tone by doping Zn^2+^ into the Ca_2_Mn_0.85_Ti_0.15_O_4_ structure

**DOI:** 10.1039/c9ra07849e

**Published:** 2019-11-28

**Authors:** Ryohei Oka, Senri Iwasaki, Toshiyuki Masui

**Affiliations:** Department of Chemistry and Biotechnology, Graduate School of Engineering, Tottori University 4-101, Koyama-cho Minami Tottori 680-8552 Japan; Faculty of Engineering, Tottori University 4-101, Koyama-cho Minami Tottori 680-8552 Japan masui@tottori-u.ac.jp +81-857-31-5264 +81-857-31-5264; Center for Research on Green Sustainable Chemistry, Tottori University 4-101, Koyama-cho Minami Tottori 680-8552 Japan

## Abstract

Inorganic black pigments with thermal barrier characteristics, Ca_2_Mn_0.85−*x*_Ti_0.15_Zn_*x*_O_4−*x*_ (0 ≤ *x* ≤ 0.10), were synthesized using a conventional solid-state reaction method in order to improve the blackness without decreasing the near-infrared (NIR) reflectance of a Ca_2_Mn_0.85_Ti_0.15_O_4_ pigment, which was previously reported by our group. The composition was optimized to provide both high blackness and NIR reflection characteristics. As a result, the NIR solar reflectance value (*R*_NIR_) of Ca_2_Mn_0.77_Ti_0.15_Zn_0.08_O_3.92_ (*R*_NIR_ = 74.6%) became larger than that of Ca_2_Mn_0.85_Ti_0.15_O_4_ (*R*_NIR_ = 71.7%), and the black color tone of the former (L* = 23.2, a* = +2.81, b* = +0.83, *C* = 2.93) was improved in comparison with that of the latter (L* = 24.4, a* = +4.30, b* = +2.72, *C* = 5.09). This improvement is caused by the introduction of strain into the [MnO_6_] octahedra and a decrease in the manganese ion concentration. The *R*_NIR_ value of the Ca_2_Mn_0.77_Ti_0.15_Zn_0.08_O_3.92_ pigment was also larger than those of the commercially available pigments (*R*_NIR_ < 53.0%). Therefore, Ca_2_Mn_0.77_Ti_0.15_Zn_0.08_O_3.92_ has potential to be an inorganic black pigment for thermal shielding.

## Abstract 2

The near infrared reflectivity and black color tone of a Ca_2_Mn_0.85_Ti_0.15_O_4_ pigment were improved by the introduction of strain with Zn^2+^ doping into the [MnO_6_] octahedra and a decrease in the manganese ion concentration.

## Graphical abstract

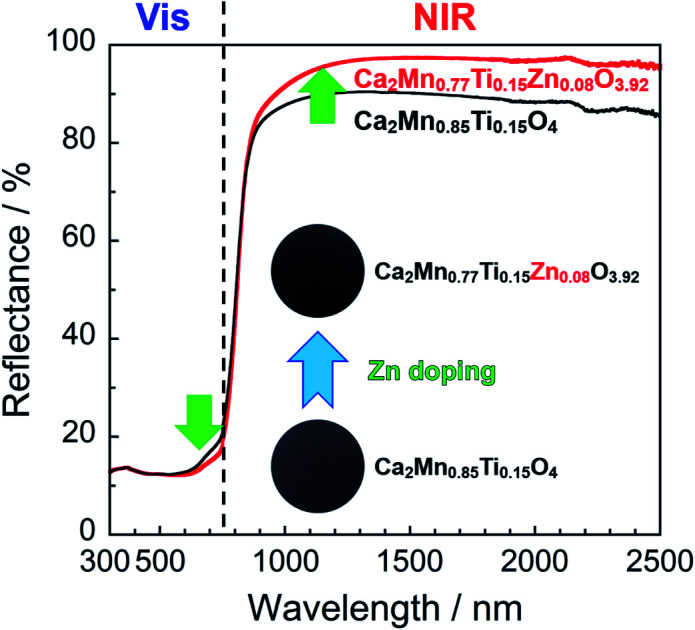

## Introduction

The urban heat-island effect leads to the ambient temperature in an urban area being higher than that in the surrounding areas.^1^ This effect often generates adverse effects such as heatstroke, discomfort, and a large consumption of electricity by air conditioners in the summer season. Natural sunlight consists of 5% ultraviolet radiation (UV; 280–400 nm), 43% visible radiation (400–700 nm) and 52% near-infrared radiation (NIR; 700–2500 nm).^2^ Since the 700–1300 nm wavelength region constitutes 80% of the total energy in the NIR region, sunlight in this range plays the most important role in generating heat.^3^ For this reason, it is effective to shield NIR light in this region in order to prevent heat storage. Many studies have been reported on several colored pigments that can reflect NIR light.^4–18^

The NIR-reflectance properties of variously colored pigments (*e.g.* white, yellow, and blue) are generally better than those of black pigments, because these pigments tend to reflect not only visible but also NIR light.^19,20^ However, black pigments such as carbon black basically absorb NIR as well as visible light to store heat. When the common black pigment on the outer walls and roofs of buildings absorbs sunlight, the temperature rises, and at the same time the amount of exhaust heat from the use of air conditioners increases. Additionally, the heat stored during the daytime is released at night, and this heat dissipation prevents night cooling. These phenomena promote the urban heat island. For this reason, application of NIR-reflective black pigments to road surfaces, building roofs, and exterior walls has attracted attention.^20,21^ Some compounds such as (Fe, Cr)_2_O_3_, Fe_2_TiO_4_, and YMnO_3_ have been proposed to serve as NIR-reflective black pigments.^21–24^ However, (Fe, Cr)_2_O_3_ contains toxic chromium, and NIR-reflective properties of Fe_2_TiO_4_ and YMnO_3_ are not enough.

In our previous study, we found that a Ca_2_Mn_0.85_Ti_0.15_O_4_ pigment was a promising novel inorganic NIR-reflective black pigment.^25^ But, unfortunately, this pigment exhibited slightly reddish black color. In this study, therefore, Zn^2+^ was doped into the Mn^4+^ site to improve the blackness without decreasing the NIR reflectance, because Zn^2+^ does not show optical absorption in the NIR region. Namely, Ca_2_Mn_0.85−*x*_Ti_0.15_Zn_*x*_O_4−*x*_ (0 ≤ *x* ≤ 0.10) samples were synthesized and the NIR reflectance and color properties were characterized. Finally, the Zn^2+^ concentration was optimized to meet both enough black hue and high NIR reflectivity.

## Experimental

### Materials and methods

The Ca_2_Mn_0.85−*x*_Ti_0.15_Zn_*x*_O_4−*x*_ (0 ≤ *x* ≤ 0.10) samples were synthesized using a conventional solid-state reaction method. Stoichiometric amounts of CaCO_3_ (FUJIFILM Wako Pure Chemical), MnO_2_ (FUJIFILM Wako Pure Chemical), TiO_2_ (FUJIFILM Wako Pure Chemical), and ZnO (Kishida Chemical) were mixed in an agate mortar. The mixtures were calcined in an alumina boat at 1200 °C for 6 h under an air atmosphere. Finally, the samples were ground in an agate mortar before characterization.

### Characterization

The samples synthesized were characterized by X-ray powder diffraction (XRD; Rigaku, Ultima IV) with Cu-Kα radiation (40 kV and 40 mA). The sampling width and the scan speed were 0.02° and 6 min^−1^, respectively. The sample compositions analyzed using X-ray fluorescence spectroscopy (XRF; Rigaku, ZSX Primus) were in good agreement with the stoichiometric compositions of the starting mixtures. The lattice parameters and volumes were calculated from the XRD peak angles, which were refined using α-Al_2_O_3_ as a standard and using CellCalc Ver. 2.20 software. The morphology of the Ca_2_Mn_0.85−*x*_Ti_0.15_Zn_*x*_O_4−*x*_ (*x* = 0 and 0.08) particles was investigated by using field-emission-type scanning electron microscopy (FE-SEM; JEOL, JSM-6701F). The size distribution and the average particle size were estimated by measuring the diameters of 200 particles from the FE-SEM photographs.

The optical reflectance spectra were measured with an ultraviolet-visible-near-infrared (UV-Vis-NIR) spectrometer (JASCO, V-770 with an integrating sphere attachment) with barium sulfate for the visible light region and polytetrafluoroethylene (PTFE) for the NIR region as references. The total (*R*_Tot_, 300–2500 nm) and NIR (*R*_NIR_, 700–2500 nm) solar reflectance was calculated in accordance with the American Society for Testing and Materials (ASTM) Standard G173-03, and was expressed as the integral of the product of the observed spectral reflectance and the solar irradiance divided by the integral of the solar irradiance, both integrated over the each range as in the formula
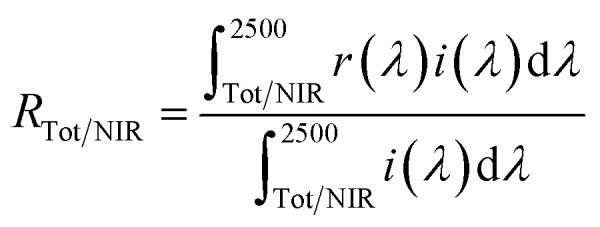
where *r*(*λ*) is the spectral reflectance obtained from the experiment and *i*(*λ*) is the standard solar spectrum (W m^−2^ nm^−1^). The color property was evaluated in terms of the Commission Internationale de l'Éclairage (CIE) L*a*b*C system using a colorimeter (Konica-Minolta, CR-300). The L* parameter indicates the brightness or darkness of a color on relation to a neutral gray scale, and the a* (the red-green axis) and the b* (the yellow-blue axis) parameters express the color qualitatively. Chroma parameter (*C*) represents the color saturation of the pigments and is calculated according to the following formula: *C* = [(a*)^2^ + (b*)^2^]^1/2^.

## Results and discussion

### X-ray powder diffraction (XRD) and field-emission-type scanning electron microscopic (FE-SEM) image

**Fig. 1:**
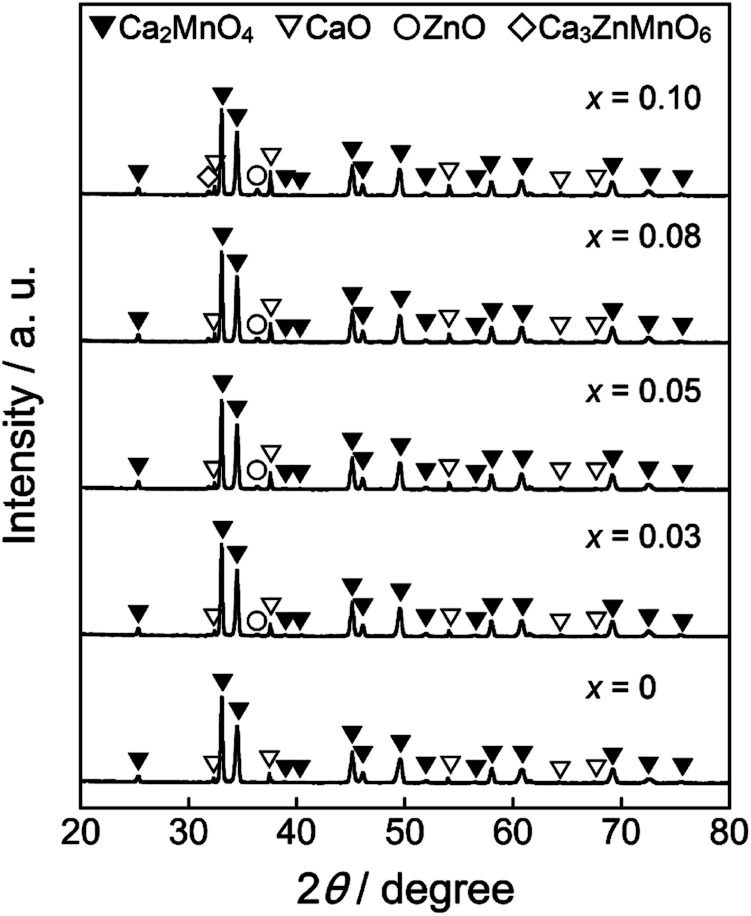
XRD patterns of the Ca_2_Mn_0.85−*x*_Ti_0.15_Zn_*x*_O_4−*x*_ (0 ≤ *x* ≤ 0.10) samples.

Fig. 1 shows the XRD patterns of the Ca_2_Mn_0.85−*x*_Ti_0.15_Zn_*x*_O_4−*x*_ (0 ≤ *x* ≤ 0.10) samples. In the *x* range from 0 to 0.08, the target phase was obtained almost in a single-phase form, although nominal CaO and ZnO phases were detected. On the other hand, a small diffraction peak indexed to Ca_3_ZnMnO_6_ was observed at 2*θ* = 32° as an additional impurity in the Ca_2_Mn_0.75_Ti_0.15_Zn_0.10_O_3.90_ (*x* = 0.10) sample.

**Fig. 2:**
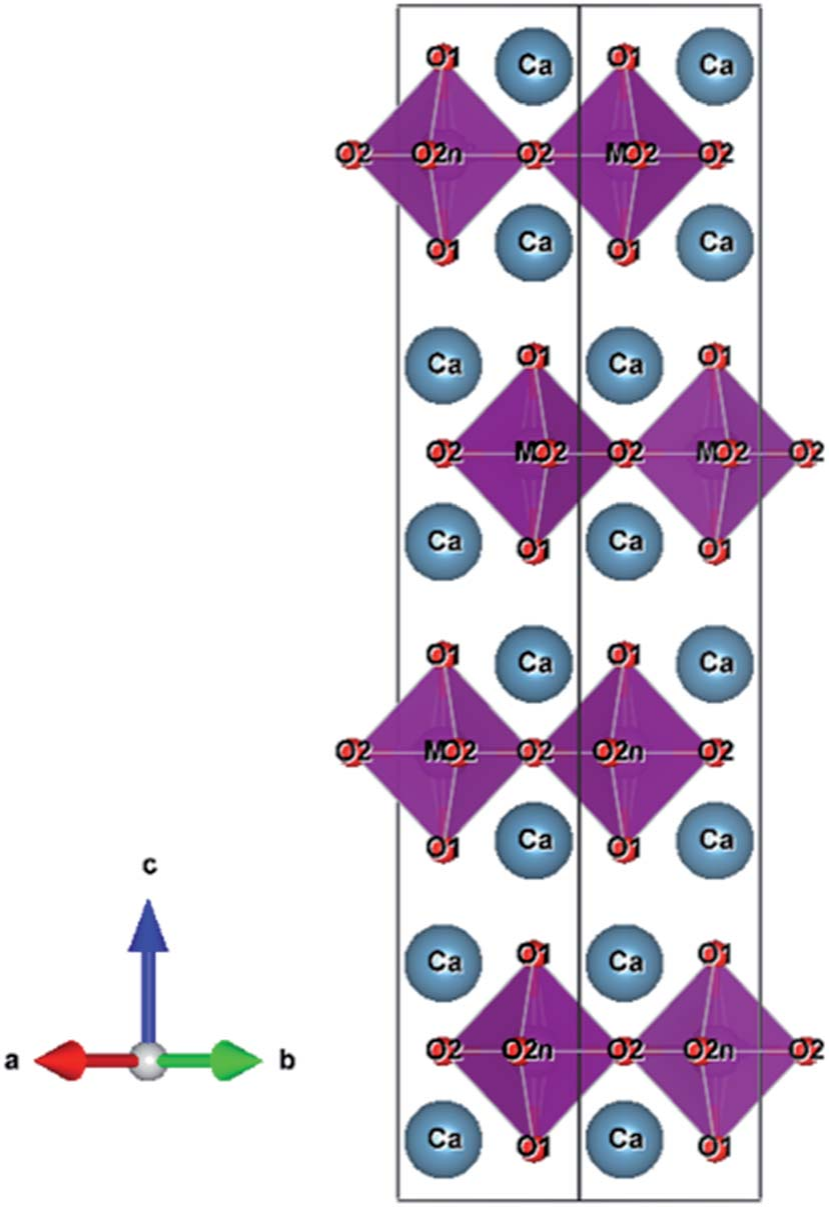
Crystal structure of Ca_2_MnO_4_.

Ca_2_MnO_4_ forms a tetragonal structure with space group of *I*4_1_/*acd* (No. 142). Fig. 2 shows the crystal structure of Ca_2_MnO_4_ illustrated using the VESTA program^27^ based on the crystallographic data reported by Leonowicz *et al.*^28^ The [MnO_6_] octahedra share corners with each other to form a two-dimensional perovskite-type array, and this [MnO_6_] layer is interleaved by CaO layers in the *c*-axis direction. The Mn^4+^ ion in the [MnO_6_] octahedron is coordinated by two O^2−^(1) ions in the *c*-axis direction and four O^2−^(2) ions on the *ab* plane. The Mn–O(1) and Mn–O(2) bond distances are 194.4 pm and 185.6 pm, respectively.^28^ Accordingly, the [MnO_6_] octahedron is tetragonally distorted.

The lattice parameters (*a*, *c*, and *V*) and the *c*/*a* ratios of all samples synthesized in this study were calculated from the XRD peak angles. These results are summarized in Table 1, where the numbers in parentheses indicate standard deviations. The cell volume increased as the Zn^2+^ concentration increased in the range of 0 ≤ *x* ≤ 0.08, indicating that some Mn^4+^ (ionic radius: 53.0 pm)^26^ ions were partially substituted with the larger Zn^2+^ (ionic radius: 74.0 pm)^26^ ions. However, the lattice volumes of Ca_2_Mn_0.77_Ti_0.15_Zn_0.08_O_3.92_ (*x* = 0.08) and Ca_2_Mn_0.75_Ti_0.15_Zn_0.10_O_3.90_ (*x* = 0.10) were equal. These results indicate that the solubility limit of Zn^2+^ was approximately *x* = 0.08 in Ca_2_Mn_0.85−*x*_Ti_0.15_Zn_*x*_O_4−*x*_.

**Table 1:** Lattice parameters (*a*, *c*, and *V*) and *c*/*a* ratio of the Ca_2_Mn_0.85−*x*_Ti_0.15_Zn_*x*_O_4−*x*_ (0 ≤ *x* ≤ 0.10) samples

*x*	*a*/nm	*c*/nm	*c*/*a*	*V*/nm^3^
0	0.52084(7)	2.4121(4)	4.6312(10)	0.6543(2)
0.03	0.52091(12)	2.4140(9)	4.634(2)	0.6550(4)
0.05	0.52096(10)	2.4159(7)	4.637(2)	0.6557(3)
0.08	0.52110(12)	2.4167(8)	4.638(2)	0.6562(4)
0.10	0.5211(2)	2.4168(12)	4.638(3)	0.6562(6)

Considering the charge compensation, the electroneutrality will be maintained by the generation of either higher valence Mn^5+/7+^ or the formation of oxide anion vacancies, when Zn^2+^ is doped into the Mn^4+^ site. However, Mn^5+^ is unstable in oxides and tends to transfer into more stable Mn^4+^ and Mn^7+^.^29^ When Mn^7+^ (ionic radius: 46 pm)^26^ is generated in the structure for charge compensation, the lattice volume should increase nonlinearly, but that is not the case. Therefore, it is reasonable to consider that the electrical neutrality of Ca_2_Mn_0.85−*x*_Ti_0.15_Zn_*x*_O_4−*x*_ is maintained by the production of oxide anion vacancies.

The lattice parameters *a* and *c* also increased with increasing the Zn^2+^ content. However, the increase rates of the former and the latter were different. The crystal structure of Ca_2_(Mn, Nb)O_4_ was investigated by Taguchi.^30^ When the Nb^5+^ ions were introduced into the Mn^4+^ site in Ca_2_MnO_4_, the *c*/*a* ratio decreased with increasing the Nb^5+^ content, and the distortion of the [MnO_6_] octahedra was alleviated.^30^ On the contrary, in the case of the Ca_2_Mn_0.85−*x*_Ti_0.15_Zn_*x*_O_4−*x*_ (0 ≤ *x* ≤ 0.10) samples synthesized in this study, the *c*/*a* ratio increased with increasing the amount of Zn^2+^ in the range of 0 ≤ *x* ≤ 0.05, as seen in Table 1. Therefore, the distortion of the [MnO_6_] octahedra was increased by the partial substitution of Mn^4+^ with Zn^2+^.

**Fig. 3:**
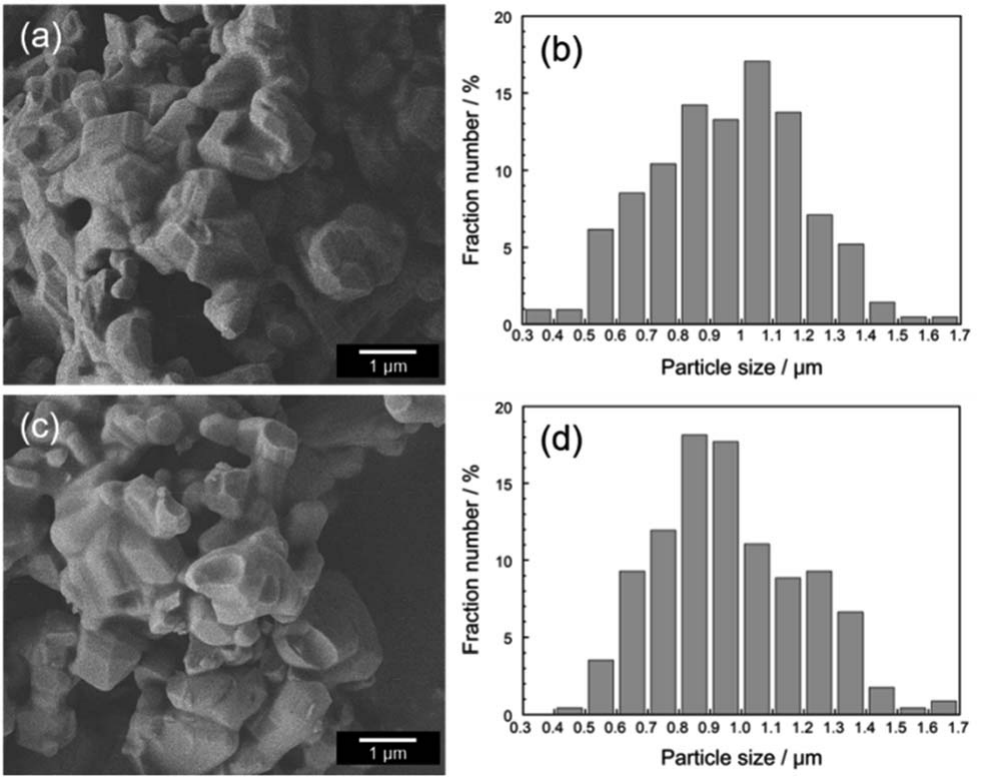
FE-SEM images and size distributions of Ca_2_Mn_0.85_Ti_0.15_O_4_ (a and b) and Ca_2_Mn_0.77_Ti_0.15_Zn_0.08_O_3.92_ (c and d).

Fig. 3 shows the FE-SEM images and size distributions of the Ca_2_Mn_0.85−*x*_Ti_0.15_Zn_*x*_O_4−*x*_ (*x* = 0 and 0.08) samples. The faceted particles were observed in both samples. These particles were thermally fused due to the high-calcination temperature at 1200 °C. In both samples, the average particle size was 0.97 μm and there was no significant change in particle size, size distribution, and morphology. These results indicate that the changes in the optical and color properties of both samples were caused by the Zn^2+^ doping.

### Reflectance spectra

**Fig. 4:**
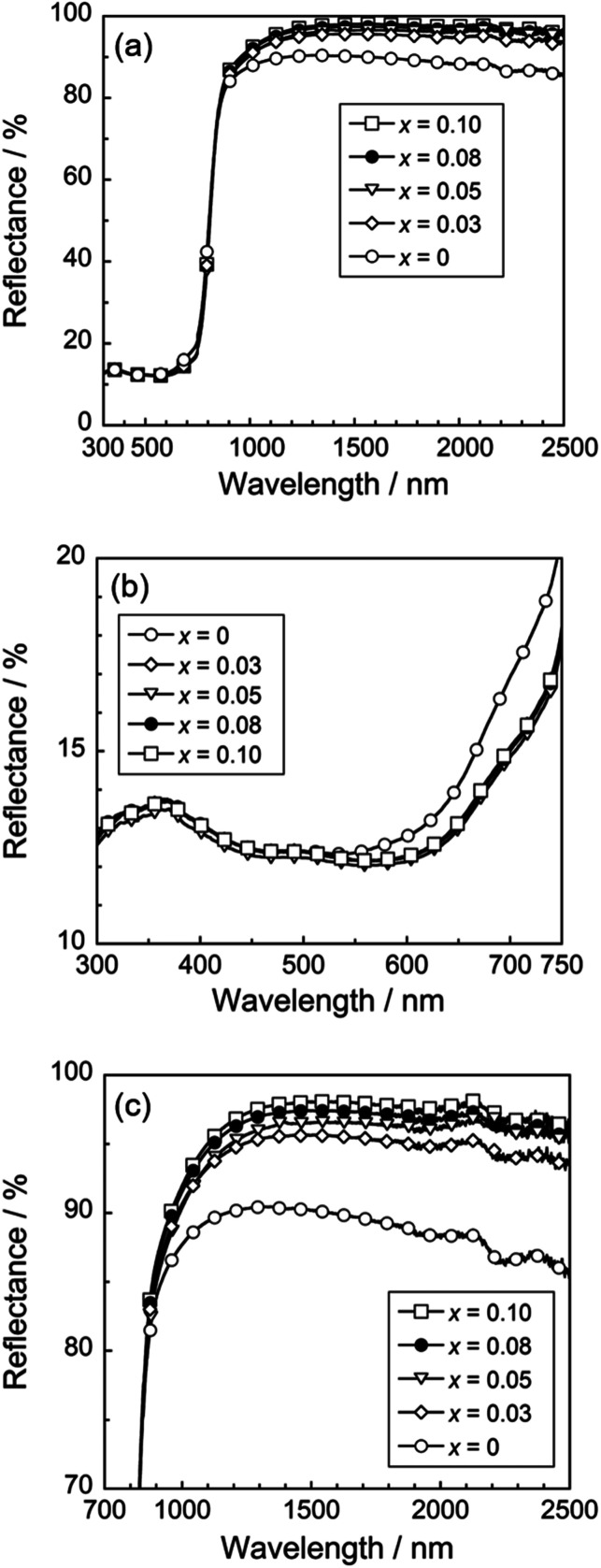
UV-Vis-NIR (a), UV-Vis (b), and NIR (c) reflectance spectra of the Ca_2_Mn_0.85−*x*_Ti_0.15_Zn_*x*_O_4−*x*_ (0 ≤ *x* ≤ 0.10) samples.

Fig. 4(a) depicts the UV-Vis and NIR reflectance spectra of the Ca_2_Mn_0.85−*x*_Ti_0.15_Zn_*x*_O_4−*x*_ (0 ≤ *x* ≤ 0.10) samples. All samples strongly absorbed visible light at a wavelength of 700 nm and shorter and reflected NIR light, due to small bandgap energies around 1.77 eV.^25^ An enlarged view of the reflectance spectra from 300 to 750 nm was shown in Fig. 4(b). Optical reflectance from 600 to 750 nm corresponding to the red light was decreased by the Zn^2+^ doping. As a result, the color of the samples changed from slightly reddish black to more vivid black and the redness of the samples was reduced.

As discussed above in Table 1, the [MnO_6_] octahedron was significantly distorted by the dissolution of Zn^2+^. This lattice distortion increased with increasing the Zn^2+^ concentration, and the symmetry of the [MnO_6_] octahedron decreased. Thus, the reduction in red light reflection was caused by the d–d transition absorption of Mn^4+^. This transition is essentially forbidden but has been partially allowed due to the loss of symmetry. On the other hand, the Mn^4+^ content of the sample was decreased by the Zn^2+^ substitution. In other words, enhancement of the optical absorption by Mn^4+^ in the red-light region and decrease of the amount of Mn^4+^ responsible for this absorption are in the relationship of trade-off. Accordingly, the optical reflectance in the red-light region almost unchanged in the range of 0.03 ≤ *x* ≤ 0.08.

On the other hand, the optical reflectance in the NIR region was increased by the Zn^2+^ doping, as seen in Fig. 4(c). The optical absorption in the NIR region was caused by the allowed charge transfer transition between Mn^4+^ and Mn^3+^ ions,^31^ and this absorption intensity depended on the concentration of manganese. Unfortunately, it is difficult to confirm how much the CaO and ZnO impurity phases are involved in improving the NIR reflectivity. Since CaO and ZnO can strongly reflect the visible light as well as the NIR light, the optical reflectance in the visible light region shall also be increased by the Zn^2+^ doping, when the effect of these impurities is large. However, the reflectance in the visible light region did not increase, but rather the reflectance of red light decreased. Therefore, the increase in the NIR reflectivity is dominantly due to the decrease in manganese ions in the sample.

### Chromatic properties and NIR solar reflectance

**Fig. 5:**
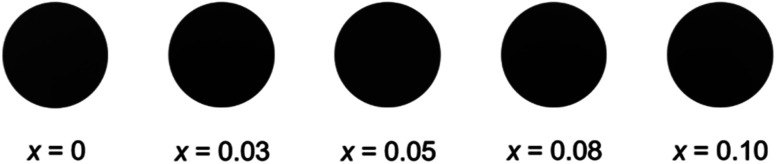
Photographs of the Ca_2_Mn_0.85−*x*_Ti_0.15_Zn_*x*_O_4−*x*_ (0 ≤ *x* ≤ 0.10) pigments.

The color coordinate data and total (*R*_Tot_) NIR solar reflectance (*R*) of the Ca_2_Mn_0.85−*x*_Ti_0.15_Zn_*x*_O_4−*x*_ (0 ≤ *x* ≤ 0.10) pigments are summarized in Table 2. The photographs of these pigments are also displayed in Fig. 5. All pigments synthesized in this study showed low L* values and were black as seen in Fig. 5. The a*, b*, and *C* values of the Zn^2+^-doped pigments were almost the same, but lower than those of the undoped Ca_2_Mn_0.85_Ti_0.15_O_4_ pigment (*x* = 0). As already discussed with respect to the results in Fig. 4(b), this was due to the decrease in the optical reflection in the red-light region (600–750 nm). The *R* value increased conversely with the Zn^2+^ doping, because the relative number of manganese ions decreased and the reflectance in the NIR region was increased as seen in Fig. 4(a) and (c).

**Table 2:** Color coordinate data and total (*R*_Tot_) and NIR (*R*_NIR_) solar reflectance of the Ca_2_Mn_0.85−*x*_Ti_0.15_Zn_*x*_O_4−*x*_ (0 ≤ *x* ≤ 0.10) pigments

*x*	L*	a*	b*	*C*	*R*_Tot_/%	*R*_NIR_/%
0	24.4	+4.30	+2.72	5.09	43.7	71.7
0.03	23.2	+2.70	+0.72	2.79	44.6	73.8
0.05	22.1	+2.84	+0.94	2.99	44.5	73.7
0.08	23.2	+2.81	+0.83	2.93	45.0	74.6
0.10	23.3	+2.97	+0.94	3.12	45.3	75.1

For an achromatic color such as black, the *C* value should be as small as possible in the L*a*b*C system. As recognized in the *C* and *R*_NIR_ values of the Zn^2+^-doped samples in Table 2, the color tone became blacker and the NIR solar reflectance was improved. Among the Ca_2_Mn_0.85−*x*_Ti_0.15_Zn_*x*_O_4−*x*_ (0 ≤ *x* ≤ 0.08) pigments synthesized in this study, Ca_2_Mn_0.77_Ti_0.15_Zn_0.08_O_3.92_ showed a relatively low *C* value and the highest *R*_NIR_ value. Therefore, it was evidenced that Ca_2_Mn_0.77_Ti_0.15_Zn_0.08_O_3.92_ has high performance as an inorganic black pigment with thermal barrier characteristics.

### Comparison with commercially available pigments

**Fig. 6:**
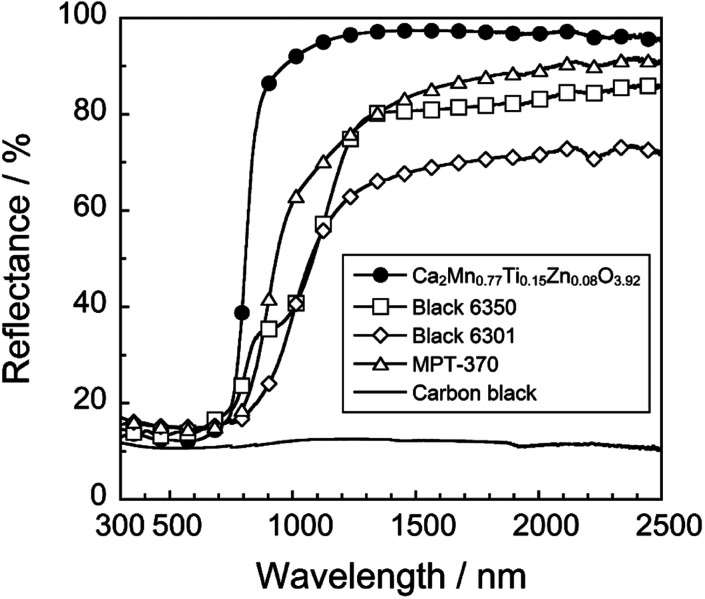
UV-Vis-NIR reflectance spectra of various black pigments.

**Fig. 7:**
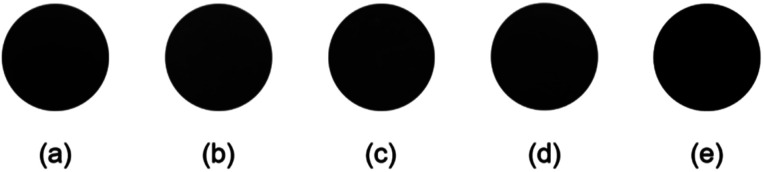
Photographs of Ca_2_Mn_0.77_Ti_0.15_Zn_0.08_O_3.92_ (a), Black 6350 (b), Black 6301 (c), MPT-370 (d), and carbon black (e).

The UV-Vis-NIR reflectance spectrum and the color parameters of the Ca_2_Mn_0.77_Ti_0.15_Zn_0.08_O_3.92_ pigment was compared with those of the commercially available black pigments such as Black 6350 (iron and chromium oxide, Asahi Kasei), Black 6301 (manganese and bismuth oxide), MPT-370 (calcium, manganese, and titanium oxide, Ca(Ti, Mn)O_3_, Ishihara Sangyo), and carbon black (Wako Chemical), as shown in Fig. 6 and summarized in Table 3. The photographs of these pigments are also displayed in Fig. 7. As evidenced from these results, the present Ca_2_Mn_0.77_Ti_0.15_Zn_0.08_O_3.92_ pigment showed higher reflectance in the NIR wavelength region and significantly higher *R*_NIR_ value than did the commercial pigments. Furthermore, the present pigment showed sufficiently low L* and *C* values, similar to the vcommercially available NIR-reflective black pigments.

**Table 3:** Color coordinate data and total (*R*_Tot_) and NIR (*R*_NIR_) solar reflectance of the black pigments

Pigment	L*	a*	b*	*C*	*R*_Tot_/%	*R*_NIR_/%
Ca_2_Mn_0.77_Ti_0.15_Zn_0.08_O_3.92_	23.2	+2.81	+0.83	2.93	45.0	74.6
Black 6350	26.4	+0.93	+4.19	4.29	31.7	47.8
Black 6301	24.0	+0.77	+1.14	1.38	28.3	40.3
MPT-370	25.1	+0.90	−0.41	0.99	34.5	52.3
Carbon black	2.69	+0.98	+1.90	2.14	11.3	11.7

### Chemical stability test

The chemical stability of the Ca_2_Mn_0.77_Ti_0.15_Zn_0.08_O_3.92_ pigment was also evaluated. The powder sample was soaked into 4% acetic acid and 4% ammonium bicarbonate aqueous solutions. After leaving them at room temperature for 24 h, the samples were washed with deionized water and ethanol, and then dried at room temperature. The NIR-reflectance properties and color of the samples after the chemical stability test was evaluated using the UV-Vis-NIR spectrometer and the colorimeter. Unfortunately, the color degradation and the decrease of the *R*_Tot_ and *R*_NIR_ values were observed by leaching the sample in both acetic acid and basic ammonium carbonate solutions, as seen in Table 4. Therefore, it is suggested that surface coating with a stable compound such as silica is necessary to suppress the deterioration.

**Table 4:** Color coordinates and total (*R*_Tot_) and NIR (*R*_NIR_) solar reflectance of Ca_2_Mn_0.77_Ti_0.15_Zn_0.08_O_3.92_ before and after chemical stability test

Treatment	L*	a*	b*	*C*	*R*_Tot_/%	*R*_NIR_/%
Non-treatment	23.2	+2.81	+0.83	2.93	45.0	74.6
4% CH_3_COOH	16.0	+10.6	+3.33	11.1	39.4	63.8
4% NH_4_HCO_3_	23.6	+4.76	+4.32	6.43	44.4	73.0

## Conclusions

Ca_2_Mn_0.85−*x*_Ti_0.15_Zn_*x*_O_4−*x*_ solid solutions were synthesized as NIR-reflective black pigments to improve the blackness of a Ca_2_Mn_0.85_Ti_0.15_O_4_ pigment without decreasing the NIR reflectance. By the introduction of Zn^2+^ in the Ca_2_Mn_0.85_Ti_0.15_O_4_ lattice, the reflection of the red-light (600–750 nm) was decreased due to the enhancement of the d–d transition of Mn^4+^, while the NIR reflectance was increased by the decrease of the charge transfer transition between Mn^4+^ and Mn^3+^. As a result, the black color tone of the non-doped Ca_2_Mn_0.85_Ti_0.15_O_4_ pigment was improved by the Zn^2+^ doping, and the highest NIR solar reflectance value (*R*_NIR_ = 74.6%) was observed at the composition of Ca_2_Mn_0.77_Ti_0.15_Zn_0.08_O_3.92_. Furthermore, the present pigment exhibited enough black color and the NIR reflectance ability is significantly higher than those of the conventional commercially available black pigments (*R*_NIR_ < 53.0%). Although it is necessary to improve the chemical stability, the Ca_2_Mn_0.77_Ti_0.15_Zn_0.08_O_3.92_ pigment has a potential to be an inorganic black pigment for thermal shielding.

## Conflicts of interest

There are no conflicts to declare.

## Supplementary Material
